# Supplementary material for: Preferences for treatment for latent tuberculosis infection in primary care among people in the United States at increased risk of tuberculosis: a pilot survey
Source: medRxiv. 2026 May 22:2026.05.20.26352199. Preprint. [Version 1] doi: 10.64898/2026.05.20.26352199 (PMC13228680; doi:10.64898/2026.05.20.26352199)
Supplement: Supplement 1 [file media-1.pdf]

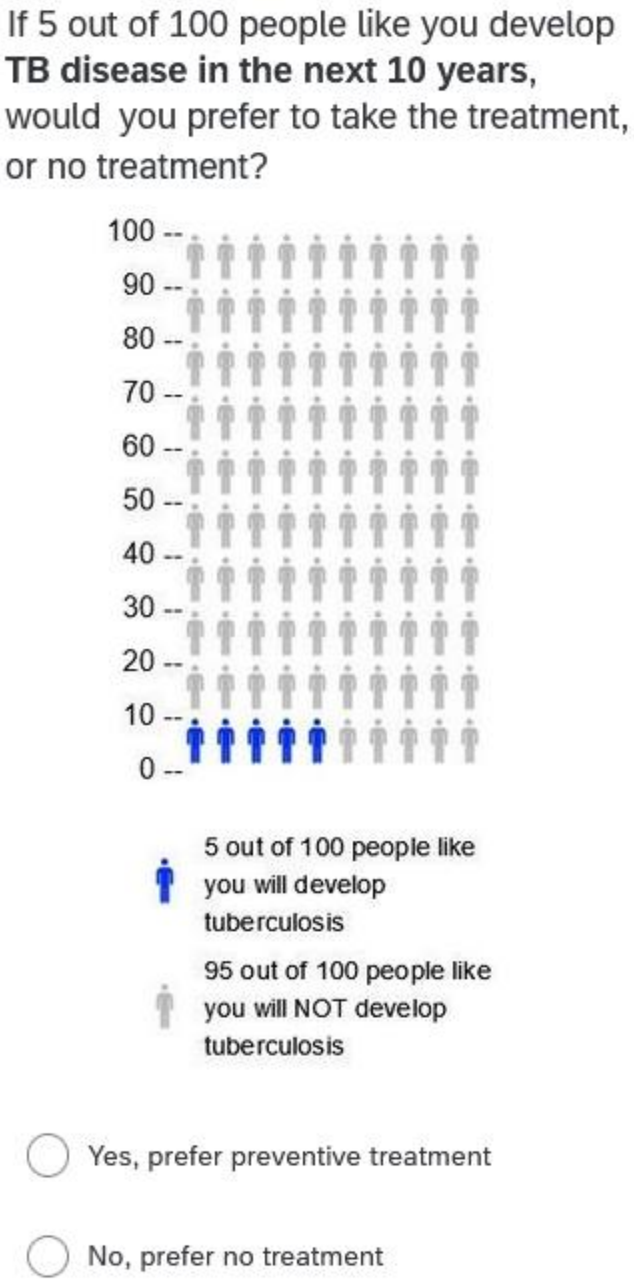

Online Supplemental Figure S1: Screenshot of an example task from the threshold exercise with a 5% risk of progression to active TB disease.
